# Application of computed tomography-guided hook-wire localization technique in thoracoscopic surgery for small pulmonary nodules (≤ 10 mm)

**DOI:** 10.1186/s13019-023-02188-3

**Published:** 2023-04-05

**Authors:** Yuan Yang, Chenhui Qin, Yue Ma, Zhongting Lu, Yun Zhang, Tao Li

**Affiliations:** 1 Graduate School of Ningxia Medical University, Yinchuan, Ningxia China; 2 Department of Surgical Oncology, Tumor Hospital, The General Hospital of Ningxia Medical University, Yinchuan, Ningxia China

**Keywords:** Hook-wire localization, CT guidance, Pulmonary nodules, Thoracoscopic surgery

## Abstract

**Objective:**

This study aimed to investigate the safety and efficacy of the computed tomography (CT)-guided hook-wire localization technique in thoracoscopic surgery for small pulmonary nodules (≤ 10 mm) and to identify the risk factors for localization-related complications.

**Methods:**

The medical records of 150 patients with small pulmonary nodules treated from January 2018 to June 2021 were retrospectively analyzed. According to preoperative hook-wire positioning status, they were divided into the localization group (50 cases) or the control group (100 cases). The operation time, intraoperative blood loss, hospital stay, and conversion rate to thoracotomy were recorded and compared between groups. Uni- and multivariate binary logistic regression analysis was used to identify the risk factors for localization-related complications.

**Results:**

A total of 58 nodules were localized in 50 patients in the localization group, and the localization success rate was 98.3% (57/58). In one case, the positioning pin fell off before wedge resection was performed. The mean nodule diameter was 7.05 mm (range, 2.8–10.0 mm), while the mean depth from the pleura was 22.40 mm (range, 5.47–79.47 mm). There were 8 cases (16%) of asymptomatic pneumothorax, 2 (4%) of intrapulmonary hemorrhage, and 1 (2%) of pleural reaction.The mean operation time of the localization group (103.88 ± 41.74 min) was significantly shorter than that of the control group (133.30 ± 45.42 min) (*P* < 0.05). The mean intraoperative blood loss of the localization group (44.20 ± 34.17 mL) was significantly lower than that of the control group (112.30 ± 219.90 mL) (*P* < 0.05). The mean hospital stay of the localization group (7.96 ± 2.34 days) was significantly shorter than that of the control group (9.21 ± 3.25 days).Multivariate binary logistic analysis showed that localization times of small pulmonary nodules in the localization group was an independent risk factor for localization-related pneumothorax.

**Conclusions:**

Our results suggest that the CT-guided hook-wire localization technique is beneficial for localizing small pulmonary nodules. Specifically, it is helpful for the diagnosis and treatment of early lung cancer because it can accurately remove lesions, decrease intraoperative blood loss, shorten operation time and hospitalization stay, and reduce thoracotomy conversion rate. Simultaneous positioning of multiple nodules can easily lead to positioning-related pneumothorax.

**Supplementary Information:**

The online version contains supplementary material available at 10.1186/s13019-023-02188-3.

## Introduction

With the spread of coronavirus disease 2019 and the popularization of low-dose spiral computed tomography (CT) in physical examination, increasing asymptomatic pulmonary nodules have been identified [1]. Pulmonary nodules [2, 3] are round or irregular lesions with a diameter of ≤ 3 cm in the lungs that appear as dense shadows on imaging. They can be well-defined or poorly demarcated lesions with single or multiple occurrences. Lesions with a diameter of 5–10 mm are defined as small pulmonary nodules. According to related study [4], the prevalence of malignant tumors among small pulmonary nodules with a diameter of 5–10 mm is 6%–28%. Therefore, a definite pathological diagnosis of small pulmonary nodules is urgently needed. However, it is challenging to determine the nature of small pulmonary nodules on imaging alone and accurately diagnose small pulmonary nodules even with the application of techniques such as fiberoptic bronchoscopy with biopsy and CT-guided fine-needle aspiration biopsy.

In recent years, thoracoscopic surgery has played an important role in the pathological diagnosis and resection of small pulmonary nodules. However, thoracoscopic surgery has limitations in the accurate resection of small pulmonary nodules, and the accurate localization of small pulmonary nodules before surgery has accordingly been the key to its success. Therefore, the clinical application of the CT-guided hook-wire localization technique in thoracoscopic surgery for small pulmonary nodules is necessary. At present, the pre- and intraoperative positioning methods in thoracoscopic surgery include CT-guided hook-wire positioning, micro-coils positioning, percutaneous injection of materials (methylene blue, agar localization, barium, lipiodol, medical glue, etc.) for positioning, intraoperative ultrasound positioning, finger palpation positioning, etc. Among them, CT-guided hook-wire positioning technology is most commonly used [5, 6]. This study aimed to investigate the practicability and safety of the clinical application of the CT-guided hook-wire localization technique in thoracoscopic surgery for small pulmonary nodules.

## Patients and methods

### Patients

The medical records of 150 patients with small pulmonary nodules treated from January 2018 to June 2021 were retrospectively analyzed. The patients were divided into the localization (50 cases) and control (100 cases) groups according to whether hook-wire small pulmonary nodule localization was performed preoperative.The control group through the chest CT and intraoperative finger palpation positioning pulmonary nodules, followed by thoracoscopic surgery.Written informed consent was obtained from all patients in advance. This study was approved by the Institutional Review Board of our institution. The work has been reported in line with the STROCSS criteria [7]. The study was conducted in accordance with the Declaration of Helsinki principles.

The inclusion criteria were as follows: small pulmonary nodules identified on CT and other imaging examinations with a maximum diameter of ≤ 10 mm; no involvement of the visceral pleura, mediastinum, or hilum; and surgical candidate with complete clinical data. The exclusion criteria were as follows: absolute contraindication for puncture and surgery; and incomplete clinical data.

### Methods

**Fig. 1:**
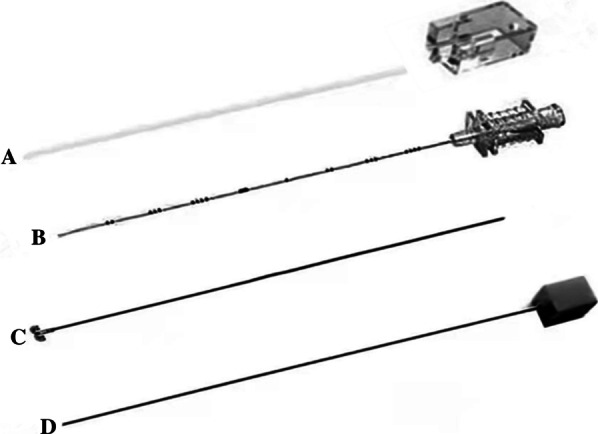
Schematic of the disposable pulmonary nodule locating needle. **a** Plastic sleeve and buckle; **b** Puncture needle; **c** Positioning line and anchor needle; **d** Push tube

A disposable pulmonary nodule locating needle (model SS510-10; Ningbo Senscure Biotechnology Co., Ltd., China) was used with an outer diameter of 0.9 mm (20 G) and a length of 10 cm. The needle consists of a plastic sleeve and buckle, puncture needle, positioning line and anchor needle, and push tube. There are scale tick marks on the positioning line, and the needle’s distal end is connected to the anchor needle. The locating needle is made of a nickel-titanium alloy, 304 stainless steel (O_6_Cr_19_Ni_10_), polyethylene terephthalate, acrylic resin and solvent, polycarbonate, and polyethylene (Fig. 1).

**Fig. 2:**
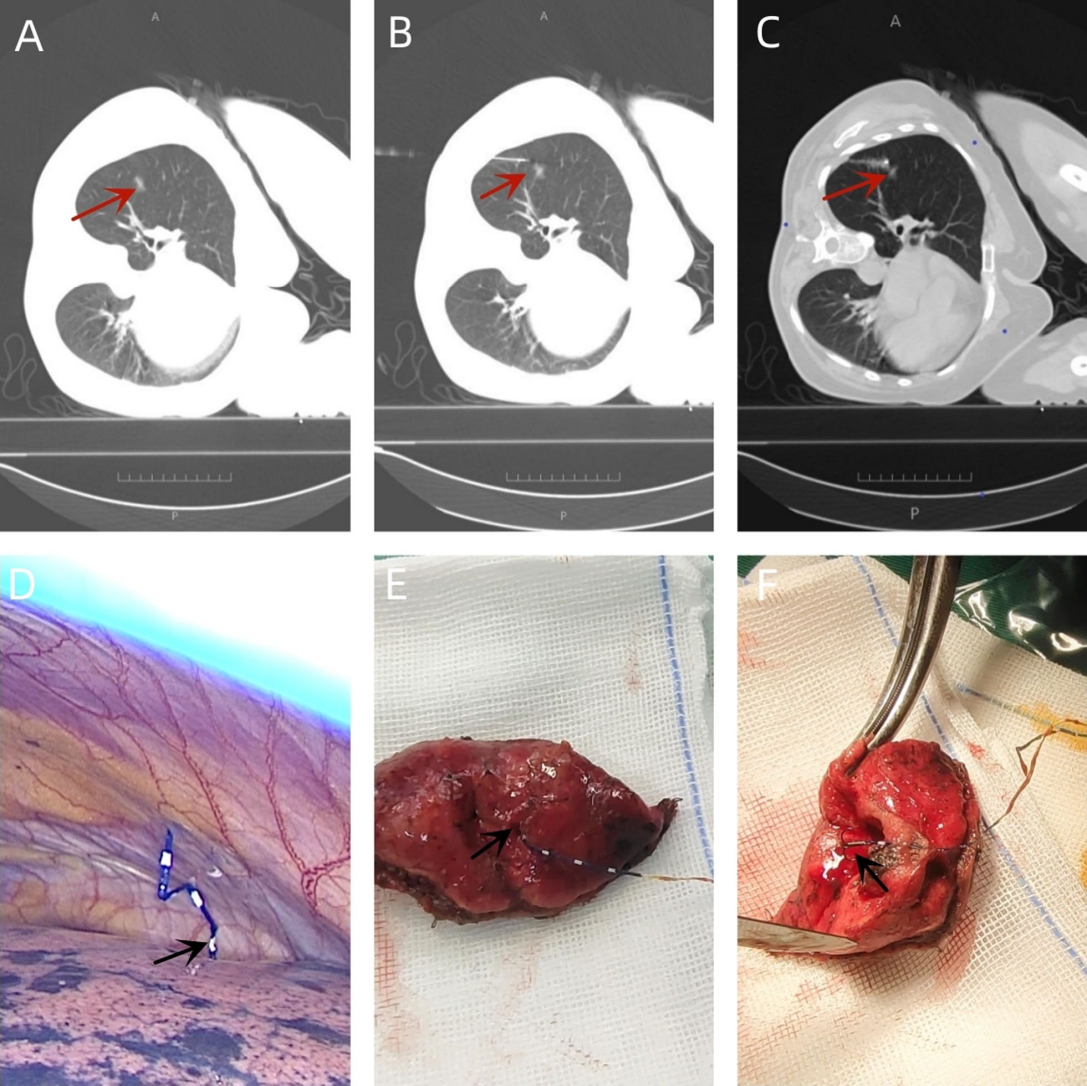
**A** The lesion with the maximum diameter. **B** Computed tomography (CT) scan to determine the accuracy of the locating needle’s position. **C** Repeat CT scan to confirm the location of the anchor needle. **D** The image under thoracoscopy after hook-wire localization. **E** The lung tissue resected by wedge resection. **F** The locating needle is in the center of the lesion

All CT-guided localization procedures were performed by skilled radiologists before surgery.Procedure was performed under local anesthesia by an experienced surgical oncologist.In the localization group, the CT-guided percutaneous hook-wire puncture positioning procedure was performed before surgery. The specific steps were as follows: 1. Imaging data and surgical plan were comprehensively analyzed, and the appropriate body position and needle entry direction were selected; 2. The body was positioned for the CT scan. According to the CT positioning results, the optimal needle insertion path, needle entry direction, and needle insertion depth were designed. The needle did not pass through the center of the lesion. The needle tip was 0.5–1 cm away from the lesion, and the needle insertion point was marked on the body surface; 3. The patient was instructed to perform breathing training. After routine disinfection, local anesthesia was performed using 2% lidocaine. A disposable pulmonary nodule locating needle was inserted along the designed path to the desired depth; 4. The CT scan was repeated. After confirming satisfactory needle insertion position and depth, the pushing device was pushed to the end and the positioning hook was released. If the puncture position was unsatisfactory, the hook was released after moderate adjustment according to the CT scan results; 5. The CT scan was repeated again. If the needle tip was determined to be within the range of 5–10 mm from the lesion, positioning was considered successful. At the same time, the pneumothorax and intrapulmonary hemorrhage were evaluated according to the CT results, and the positioning line was pushed out of the needle tip to the pleural cavity. Next, the puncture needle and the pushing device were pulled out of the body, and the wound was bandaged with sterile gauze; 6. The positioning timing started from when the body was ready for CT scanning, while the time for the repeat CT scan after the positioning was completed was recorded as the end time and patients underwent video-assisted thoracoscopic (VATS) surgery on the same day; 7. The surgical procedure was performed using a uni- or biportal technique. The nodules were localized based on their proximity to the positioning line and the resection depth was guided according to the hook position. Wedge resection of the nodules was performed, specimens were sent for pathological examination. When frozen specimens are carcinoma in situ, minimally invasive adenocarcinoma, atypical adenomatous hyperplasia, benign lesions are not further removed. When the frozen specimen is Minimally Invasive Adenocarcinoma, lymph node dissection is performed on the basis of the first resection. When the frozen specimen is Invasive adenocarcinoma, lobectomy and mediastinal lymph node dissection are performed with VATS if physiologically feasible;8. In the control group, intraoperative observation or finger palpation were used to locate the pulmonary nodules, the hook-wire positioning under CT guidance was not performed, and the rest of the steps were as described above (Fig. 2).

### Statistical analysis

All statistical analyses were carried out with spss25.0 software. Measurement data was shown as mean ± standard deviation (SD).Independent-samples t-test was used for data conforming to normality. Otherwise, Wilcoxon sign rank sum test was used. Binary logistic regression analysis was used for univariate and multivariate analysis. A *P*-value ≤ 0.05 was considered statistically significant.

## Results

A total of 50 cases in the localization group (58 nodules) underwent CT-guided hook-wire localization for puncture. Of them, 57 lesions (98.3%) were successfully located, while localization failed in one (1.7%). The patients included 13 men (26%) and 37 women (74%) aged 28–73 (mean, 54.66 ± 9.373) years old. The simultaneous localization of two pulmonary nodules was performed in eight patients; the mean nodule diameter was 7.046 ± 2.248 (range, 2.8–10.0 mm) mm. Ten patients (20%) had a history of smoking and six (12%) had a history of lung disease. Nine lesions (15.5%) were solid, 41 (70.7%) were pure ground glass nodules, and eight (13.8%) were part-solid nodules.

Twenty-two lesions (37.9%) were in the right upper lobe of the lung, 12 (20.7%) were in the right lower lobe, seven (12.1%) were in the right middle lobe, 11 (18.9%) were in the left upper lobe, and six (10.3%) were in the left lower lobe. The postoperative pathology was malignant in 68.9% of the lesions. There were 100 patients (35 men [35%], 65 women [65%]) in the control group aged 29–77 (mean, 54.75 ± 10.032) years. Simultaneous resection of two ipsilateral small pulmonary nodules was performed in 10 patients. The mean nodule diameter was 7.795 ± 2.055 (range, 3–10.0) mm. There were 13 (11.8%) solid nodules, 81 (73.6%) pure ground glass nodules, and 16 (14.6%) mixed ground glass nodules. A total of 32 lesions (29.1%) were in the right upper lobe of the lung, 21 (19.1%) were in the right lower lobe, 12 (10.9%) were in the right middle lobe, 26 (23.6%) were in the left upper lobe, and 19 (17.3%) were in the left lower lobe. The postoperative pathology of 76.3% of the nodules was malignant. There was no significant intergroup differences in the general data (*P* ≥ 0.05) (Table 1).

**Table 1:** Patients’ general characteristics by study group

	Localization group	Control group	*P*
Number of patients (n)	50	100	
Sex, n (%)			0.265
Male	13 (26)	35 (35)	
Female	37 (74)	65 (65)	
Mean age, years, $\overline{x}$ ± s	54.66 ± 9.373	54.75 ± 10.032	0.734
History of lung disease, *n* (%)			0.473
Positive	6 (12)	7 (7)	
Negative	44 (88)	93 (93)	
History of smoking, *n* (%)			0.767
Positive	10 (20)	18 (18)	
Negative	40 (80)	82 (82)	
Nodule type, *n* (%)	58	110	0.286
Single occurrence	42 (84)	90 (90)	
Multiple occurrences	8 (16)	10 (10)	
Nodule diameter, mm, $\overline{x}$ ± s	7.046 ± 2.248	7.795 ± 2.055	0.053
Nodule properties, n (%)			0.796
Solid nodules	9 (15.5)	13 (11.8)	
Pure ground glass nodules	41 (70.7)	81 (73.6)	
Mixed ground glass nodules	8 (13.8)	16 (14.6)	
Nodule location, *n* (%)			0.623
Right upper lobe	22 (37.9)	32 (29.1)	
Right middle lobe	7 (12.1)	12 (10.9)	
Right lower lobe	12 (20.7)	21 (19.1)	
Left upper lobe	11 (19.0)	26 (23.6)	
Left lower lobe	6 (10.3)	19 (17.3)	
Type of final surgical approach after pathological diagnosis, n (%)			0.056
Pulmonary wedge resection	38 (76)	56 (56)	
Segmentectomy	6 (12)	24 (24)	
Lobectomy	6 (12)	20 (20)	
Postoperative pathology, n (%)			0.282
Adenocarcinoma in situ	27 (46.6)	49 (44.5)	
Invasive adenocarcinoma	10 (17.2)	30 (27.3)	
Minimally invasive adenocarcinoma	3 (5.2)	5 (4.5)	
Atypical adenomatous hyperplasia	6 (10.3)	9 (8.2)	
Benign lesions	12 (20.7)	17 (15.5)	

$\overline{x}$ ± s shown as mean ± standard deviation, *n* (%) means number (proportion)

### CT-guided localization and puncture outcomes

There were 50 patients in the localization group, in which a total of 58 pulmonary nodules were localized. The localization success rate was 98.3% (57/58). The reason for the failure to locate one pulmonary nodule was that it was found to fall off during surgery after successful localization. Finally, according to the location of the bleeding point of the puncture needle, the video-assisted thoracoscopic wedge resection of the lung was successfully performed. The mean puncture depth was 54.89 ± 13.915 (range, 30.56–84.8) mm, and the mean puncture time was 14.66 ± 5.181 (range, 10–35) min. The incidence of localization-related complications was 24% (12/50), while the incidence of pneumothorax was 16% (8/50); all were asymptomatic pneumothorax requiring no specific treatment. The incidence of intrapulmonary hematoma was 4% (2/50); no relevant treatment was adopted since the patient did not complain of discomfort after positioning. There was one case (2%) of pleural reaction that was relieved after rest; no special treatment was provided. One patient (2%) successfully underwent wedge resection of the lung according to the hematoma at the puncture point on the lung surface and the localization depth after unhooking of the locating needle. No serious complications such as severe pneumothorax, massive hemorrhage, or embolism occurred during the puncture process (Table 2).

**Table 2:** Computed tomography-guided localization and puncture outcomes

	Value
Nodule diameter, mm	7.05 ± 2.248 (2.8–10.0)
Distance from pleura, mm	22.40 ± 11.395 (5.47–79.47)
Puncture depth, mm	54.89 ± 13.915 (30.56–84.8)
Puncture time, min	14.66 ± 5.181 (10–35)
Single occurrence	13.31 ± 2.542 (10–18)
Multiple occurrences	22.75 ± 7.924 (15–35)
Localization-related complications	12 (24.0)
Pneumothorax	8 (16.0)
Intrapulmonary hematoma	2 (4.0)
Pleural reaction	1 (2.0)
Half-shedding	1 (2.0)

Values are shown as mean ± standard deviation (range) or *n* (%)

### Surgery-related indicators of patients by group

The mean operation time of the localization group was 103.88 ± 41.72 min, which was significantly shorter than that of the control group (133.30 ± 45.42 min, *P* < 0.05). The mean intraoperative blood loss (44.20 ± 34.17 mL) was significantly lower in the localization group than in the control group (100.60 ± 132.78 mL, *P* < 0.05). The mean days of hospitalization was significantly shorter in the localization (7.96 ± 2.34 days) than in the control group (9.21 ± 3.25 days, *P* < 0.05) (Table 3).

**Table 3:** Surgery-related indicators by study group ($\overline{x}$ ± s)

	Localization group (*n* = 50)	Control group (*n* = 100)	*P*
Operation time, min	103.88 ± 41.72	133.30 ± 45.42	0.000
Intraoperative blood loss, mL	44.20 ± 34.17	112.30 ± 219.90	0.000
Hospital stay, days	7.96 ± 2.34	9.21 ± 3.25	0.025

### Uni- and multivariate analysis of localization-related pneumothorax

In our attempt to further clarify the risk factors for localization-related pneumothorax in the localization group, three variables were identified as likely associated with localization-related pneumothorax through univariate analysis. In the multivariate logistic regression analysis, the localization times of nodules was an independent risk factor for localization-related pneumothorax. (odds ratio, 10.966; 95% confidence interval, 1.333–90.236; *P* = 0.026) (Table 4).

**Table 4:** Univariate and multivariate analyses of localization-related pneumothorax

	Univariate analysis		Multivariate analysis	
	OR (95% CI)	*P*	OR (95% CI)	*P*
Age	0.951 (0.882–1.026)	0.192		
Sex	1.920 (0.388–9.489)	0.424		
History of smoking	6.000 (1.172–30.725)	0.032	2.341 (0.287–19.108)	0.427
History of lung disease	7.800 (1.225–49.677)	0.030	4.136 (0.450–38.056)	0.210
Localization time	0.719 (0.502–1.028)	0.071		
Puncture depth	0.975 (0.918–1.036)	0.412		
Localization times of nodules	21.667 (3.402–137.992)	0.001	10.966 (1.333–90.236)	0.026
Distance from pleura	0.890 (0.792–1.000)	0.050		
Nodule diameter	1.284 (0.879–1.876)	0.197		

*CI* confidence interval; *OR* odds ratio

### Reasons for conversion to thoracotomy and lobectomy

In the control group, 16 cases (16%) were converted to thoracotomy and lobectomy during surgery (Table 5). The main reason for the conversion to lobectomy was that the finger palpation did not touch the pulmonary nodules during the operation (11 cases) and the conversion thoracotomy was the discovery of severe pleural adhesions (5 cases). One case (2%) in the localization group was converted to thoracotomy due to severe pleural adhesions. The conversion rate to thoracotomy and lobectomy was 2% in the localization group versus 16% in the control group (Table 5).

**Table 5:** Reasons for conversion to thoracotomy and lobectomy by study group

	Localization group	Control group
Failed preoperative localization	–	–
Decoupling	–	–
Failed intraoperative localization	–	11
Severe pleural adhesions	1	5
Total converted to thoracotomy and lobectomy	1 (2%)	16 (16%)

## Discussion

In recent years, the incidence of lung cancer has been second only to breast cancer; however, it still has the highest mortality rate [8]. With the increasing popularity of low-dose spiral CT in physical examination, the detection rate of small pulmonary nodules has increased significantly. Approximately 50% of small pulmonary nodules are malignant [9]. However, it is difficult to identify the pathology using traditional CT-guided percutaneous lung puncture and bronchoscopy with biopsy, and false-negative results occur. According to relevant reports [10, 11], in 29% of cases, the possibility of malignancy cannot be ruled out due to insufficient histological evidence. VATS has the advantages of a high diagnostic rate, short operation time, and minimal trauma; thus, it has become an important method for the diagnosis and treatment of small pulmonary nodules [11]. However, VATS has limitations in terms of resection of small pulmonary nodules due to the small size of the pulmonary nodules and difficulty with lesion localization. As a result, accurate positioning has become the key to the diagnosis and treatment of small pulmonary nodules. Multiple positioning methods used in the clinical setting can be broadly divided into two categories: intraoperative non-invasive localization and preoperative invasive localization. Among them, the common non-invasive localization methods include intraoperative finger palpation and intraoperative ultrasound localization.

Invasive localization methods include CT-guided hook-wire localization, CT-guided percutaneous injection of material (micro-coil positioning, methylene blue, agar positioning, barium, lipiodol, medical glue, etc.) for positioning and other positioning methods.

In recent years, relevant medical practitioners have used electromagnetic navigation bronchoscopy (ENB) for intraoperative localization. Awais and Luo et al [12, 13] confirmed the successful application of ENB in the localization and resection of pulmonary nodules, Chao [14] et al. performed intraoperative localization in a hybrid operating room (HOR), which has a similar diagnostic rate compared to other invasive preoperative localization such as percutaneous lung puncture localization. The rates of complications such as pneumothorax and bleeding was also significantly reduced [15]. However, ENB has high technical requirements for operation, high costs of mixed laboratories, and expensive inspection equipment. Only a few hospitals in China have such equipment and mixed operating rooms, which limits its clinical popularity.

It is generally believed that the CT-guided hook-wire localization technique is the most commonly used pulmonary nodule localization technology [5, 6, 9]. This localization technique requires the placement of an anchor needle (first used in the localization of breast lumps) in the lung tissue adjacent to the pulmonary nodule under CT guidance. This locating needle is a disposable pulmonary nodule locating needle improved by Fan [16] et al. based on the traditional hook-wire positioning device. The most common complication of this positioning technique is pneumothorax. Hanauer [17] et al. studied 181 cases of solitary pulmonary nodules and found that the rate of pneumothorax in patients underwent hook-wire localization could reach 38%. The incidence of pneumothorax in this study was 16% (8/50), which was lower than that reported in the above study. This may be related to the skilled operation of the surgeons and their mastery of the indications for preoperative hook-wire puncture positioning. Upon studying 276 patients with hook-wire positioning, Iguchi et al. [18] concluded that increased respiratory motion during positioning, pulmonary nodules that are located in the lower lobe of the lung, pulmonary nodules with solid components, prone patient position, and puncture path that passes through the interlobar fissures are all factors that may cause pneumothorax.

Most patients have mild symptoms that do not require special treatment. Li et al. [19] reported that the incidence of hook-wire pulmonary hemorrhage was 13.9%–36%. In this study, the incidence of intrapulmonary hemorrhage was 4% (2/50), which is much lower than those reported in the above-mentioned studies. This may be related to the small number of cases in the localization group. Hwang et al. [20] performed hook-wire localization in 45 patients and found that hook-wire displacement occurred in 8.9% of patients. In our study, half-shedding occurred only in one patient (2%) after positioning. The displacement rate was much lower than that reported in the other hook-wire studies. In the one case in which the locating needle fell off in this study, the lesion was relatively close to the pleura. The displacement of the locating needle is related to the depth of the release position of the puncture needle and preoperative pulling to the locating needle. Pleural reactions are generally caused by irritation of the pleura by detachment of the positioning line or the locating needle, which is common in elderly and frail patients. In this patient, the occurrence of pleural reaction was due to his excessive tension and weakness, leading to dizziness and a decrease in blood pressure after the puncture; his symptoms were relieved after rest, and no serious complications occurred. However, in clinical practice, it is necessary to prevent the occurrence of severe pleural reactions.

In the diagnosis and treatment of small pulmonary nodules, CT-guided hook-wire localization technology can reportedly [21] help accurately locate and rapidly remove lesions during thoracoscopic surgery, thus effectively shortening the operation time. The duration of thoracoscopic surgery, intraoperative bleeding and hospital stay of patients in both groups were recorded, and the results showed that the operation time, intraoperative bleeding and hospital stay in the positioning group were significantly lower than those in the control group, which is consistent with the results of the above studies.

This study explored the efficacy and safety of CT-guided hook-wire localization in thoracoscopic surgery for small pulmonary nodules through clinical practice with a certain number of cases. The univariate analysis revealed that three variables were associated with localization-related pneumothorax. The number of localized small pulmonary nodules was an independent risk factor for localization-related pneumothorax, as was indicated by multivariate regression analysis, consistent with previous findings [22]. Considering that relatively more holes were created by the insertion of multiple locating needles into the visceral pleura due to the simultaneous placement of two locating needles in our study, the chance of air entering the pleural cavity was increased [23]. In the course of clinical diagnosis and treatment, many patients have multiple occurrences of pulmonary nodules. The incidence of pneumothorax in patients during localization process may be relatively high, so effective measures to prevent pneumothorax should be prepared before the procedure. The rate of conversion to thoracotomy in the two groups was 2% versus 16% (control versus localization group), and the difference was statistically significant. In this study, 68.9% and 76.3% of the resected small pulmonary nodules in the localization group and the control group, respectively, were finally diagnosed as malignant; both were higher than the previously reported malignancy rate of small pulmonary nodules with a diameter of < 10 mm (6%–28%); this may be due to the fact that only those small pulmonary nodules that are highly suspected as malignant by the observation of their imaging properties are further inspected surgically for diagnosis and treatment in clinical practice; no further analysis would be performed because the localization was not necessarily related to the postoperative nature of the nodules. Once the hook-wire localization technique guided by CT is practiced in a large number of cases, it can be developed into a localization technique that is mastered by physicians in clinical departments. This can significantly improve the early diagnosis and resection rates of small pulmonary nodules and provide effective help for the detection and treatment of early-stage lung cancer (Additional file 1).

This retrospective study was performed at a single center with a small sample size. Thus, future multicenter studies with large sample sizes are needed to validate our findings.

In conclusion, CT-guided hook-wire localization technique has certain clinical application value in thoracoscopic surgery for small pulmonary nodules. It can accurately locate small pulmonary nodules, reduce intraoperative blood loss, shorten operation time and hospitalization days, and reduce the conversion rate to thoracotomy, thereby facilitating the early diagnosis and treatment of lung cancer. Simultaneous localization of multiple nodules can easily lead to the occurrence of localization-related pneumothorax. Thus, this technique is worth spreading.

## Supplementary Information

**Additional file 1. **Application form for ethical review

## Data Availability

The data that support the findings of this study are available from the corresponding author, [author initials], upon reasonable request.
