# Supplementary figures and images for: Application of computed tomography-guided hook-wire localization technique in thoracoscopic surgery for small pulmonary nodules (≤ 10 mm)
Source: J Cardiothorac Surg. 2023 Apr 5;18:99. doi: 10.1186/s13019-023-02188-3 (PMC10074372; doi:10.1186/s13019-023-02188-3)

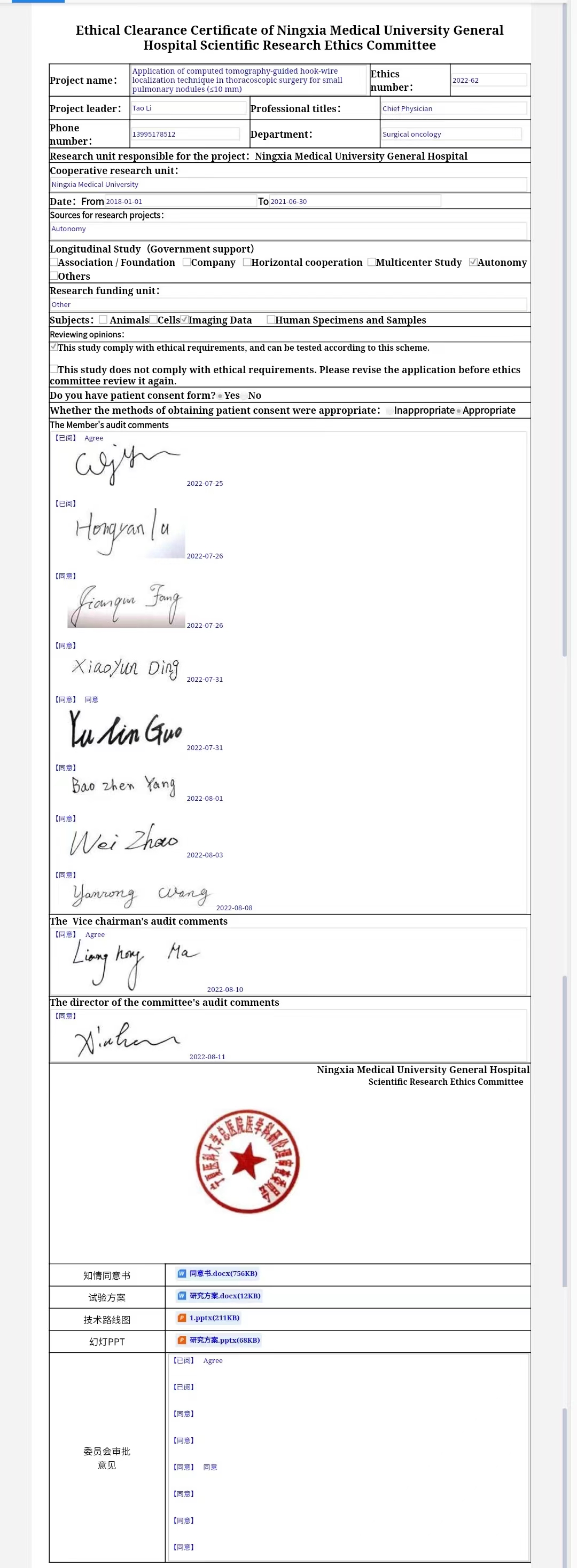

Supplement: Supplementary file 1 — Additional file 1. Application form for ethical review [file 13019_2023_2188_MOESM1_ESM.jpg]
